# Synthesis of Nanocomposite TiSiCN Coatings by Titanium Evaporation and Organosilicon Compound Activation in Hollow Cathode Arc Discharge

**DOI:** 10.3390/membranes12030321

**Published:** 2022-03-12

**Authors:** Andrey I. Menshakov, Yulia A. Bruhanova, Andrey I. Kukharenko, Ivan S. Zhidkov

**Affiliations:** 1 Institute of Electrophysics of the Ural Branch of the Russian Academy of Sciences, 106 Amundsen Street, 620016 Yekaterinburg, Russia; yuliya.bryuhanova.96@mail.ru; 2 Institute of Physics and Technology, Ural Federal University, 19 Mira Street, 620002 Yekaterinburg, Russia; a.i.kukharenko@urfu.ru (A.I.K.); i.s.zhidkov@urfu.ru (I.S.Z.); 3 M.N. Mikheev Institute of Metal Physics, Ural Branch of the Russian Academy of Sciences, 18 Sofia Kovalevskoi Street, 620990 Yekaterinburg, Russia

**Keywords:** TiSiCN, nanocomposite coatings, PVD, PECVD, hollow cathode arc, anodic evaporation

## Abstract

TiSiCN coatings have been obtained by anode evaporation of titanium and the decomposition of hexamethyldisilazane in an arc discharge, using a self-heated hollow cathode, at the pressure rate of 1 mTorr of the Ar+N_2_ gas mixture. The proposed method makes it possible to independently and widely change the amount of metal and precursor vapor flows, the pressure and composition of the vapor-gas mixture and the degree of ionic interaction on the surface of the growing coating within a single discharge system. The paper presents the method and the results of the effect of a current discharge (10–50 A), and the flux of precursor vapours (0–1 g/h), on deposition rates, compositions, and properties of TiSiCN coatings deposited by an advanced combined PVD+PECVD method. Dense homogeneous TiSiCN coatings up to 6 µm thick and up to 27.5 GPa in hardness were obtained at 7.5 µm/h. The composition of the obtained coatings has been studied by X-ray diffraction and X-ray photoelectron spectroscopy, and it has been shown that the presented methods can form nanocomposite coatings with nanocrystallites TiC, TiN, and TiC_x_N_1−x_ 3–10 nm in the amorphous matrix based on SiCN.

## 1. Introduction

Nanocomposite (nc) TiSiCN coatings are one of the most promising protective wear-resistant coatings due to their unique properties. The advantage of this nc structure, usually consisting of nano-dimensional Ti (C, N) crystallites embedded in the amorphous Si_3_N_4_/SiC [1], is the high thermal resistance that allows TiSiCN coatings to maintain a high hardness of up to 800 °C [2], as well as oxidation resistance up to 900 °C [3]. Moreover, nc-TiSiCN coatings have superior antifriction properties due to the presence of amorphous or diamond-like carbon in certain areas [4,5]. Owing to the combination of self-lubricating properties, high hardness and high impact strength, TiSiCN coatings can provide better durability than conventional nitride or carbonite coatings [6]. TiSiCN also shows a 5–10 times higher resistance to dust erosion than many nitride coatings [7], which makes them promising for protecting the blades of gas-turbine engines. These titanium-based coatings also have high bio-compatibility, making them attractive in the field of bio-medicine [8] that requires, in addition to high wear resistance, high adhesion to titanium alloys implants. The conditions and the method of their synthesis have a certain effect on the properties of coatings.

The most conventional methods of producing TiSiCN are various modifications of cathode arc evaporation of titanium [9,10] as well as magnetron sputtering of titanium and multi-component targets [11], CVD methods [12], including plasma-enhanced chemical vapour deposition (PECVD) methods, based on the use of liquid organosilicon precursors (OSCs) [7,10]. Such precursors are used because of their low cost and fairly wide range of OSCs with various relative contents of Si, C, N components. The applied precursors already have Si–C and Si–N bonds that are necessary to form TiSiCN coatings. Therefore, in order to facilitate the formation of films containing these bonds, it is important to control the decomposition rate of initial molecules. A usual process of magnetron sputter is known to frequently cause a porous microstructure due to a low ionisation degree of target particles [13], so additional plasma generators are usually used to improve current density [14]. Moreover, deposition rates during magnetron sputtering are relatively low, and the use of multi-component targets makes the optimisation of the elemental composition of coatings too time-consuming. CVD method of TiSiCN coatings deposition in a gas mixture containig TiCl_4_, SiH_4_ and other components [12] allows to obtain coatings with HV of up to 40–41 GPa at a sufficiently high deposition rate (4 µm/h). However, firstly, this process takes place at sufficiently high temperatures (800–900 °C), and another negative factor limiting its applicability is that toxic gases are used as reagents, which are environmentally hazardous substances harmful to human health. Vacuum arc methods provide high deposition rates, but it is hard to eliminate micro-particles and porosity formed in the coatings [15,16]; using microdrop vapour filtration systems results in a significant reduction in deposition rate and ion current density.

A discharge with a self-heated hollow cathode or hollow cathode arc (SHHC, HCA) [17] and active anode seem attractive for producing multi-component coatings. In such a discharge, a dense plasma is created in a large volume without the use of a separate vapour-gas ionization source, and no filtration systems are applied since the flow of the evaporated metal contains no drops. This approach was used for producing binary a-Al_2_O_3_–coatings by Al evaporation in the oxygen-containing plasma [18] and TiN-coatings by Ti evaporation in nitrogen plasma [19]. The HCA was successfully applied to produce SiCN coatings and was efficient in the activation of hexamethyldisilazane ([(CH_3_)_3_Si]_2_NH, HMDS) vapours [20].

The present paper proposes and describes a method, the main advantage of which is that the formation of a stream of titanium vapours, a dense plasma, and the activation of precursor vapours occurs in one discharge cell, wherein the density of ion current on the surface of the samples, the flow of precursor vapours and the rate of evaporation of the metal can vary independently in broad ranges using a separate anode crucible and ioniser. In this way, drop-free plasma is created without the use of additional filtration systems and separate devices to increase plasma density and provide metal melting. The possibility of independent changes in almost all processing conditions allows us to investigate the influence of these factors on the composition and properties of the coatings obtained and subsequently obtain films with the required characteristics.

The results are presented of the conditions for producing TiSiCN-coatings by anode titanium evaporation and the decomposition of HMDS vapours in HCA discharge. The results are also given of the study of the effect of titanium and HMDS vapour on the voltage discharge characteristics and plasma composition, the measurement of the deposition rate, hardness, composition and structure of TiSiCN coatings which depend on the discharge current.

## 2. Materials and Methods

**Figure 1:**
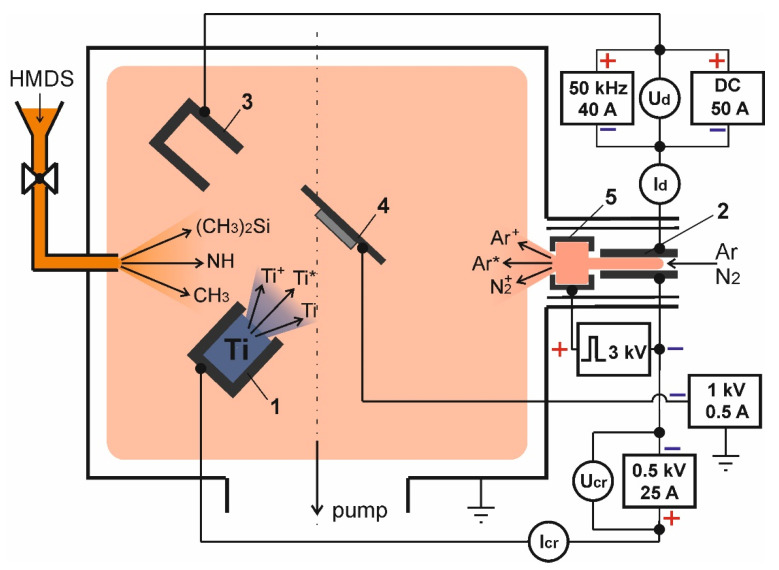
Electrode scheme of experimental facility.

The electrode system with its supply diagram is shown in Figure 1. The experimental setup is based on a gas-discharge system with an SHHC similar to [21]. In this work, the discharge is used not only to ionize the gas and decompose the precursor vapours, as in [20], but also to heat the crucible 1 and titanium evaporation using two independent anode sections. A tubular cathode 2 with a length of 70 mm and an internal diameter of 6 mm made of TiN powder by magnetic-impulse pressure was used [22]. The segmented anode consisted of a water-cooled section 3 of stainless steel and a non-cooled crucible of MPG-7 graphite. The crucible was charged with VT1–0 titanium at 0.4–0.6 g. To improve heat insulation, the crucible was placed into a heat shield of molybdenum and a ceramic tube so that the electrical current was circuited only to the open face surface of the crucible, which also ensured increased specific capacity of the discharge and more efficient heating of the emission surface.

HMDS ([(CH_3_)_3_Si]_2_NH) was selected as an organosilicon precursor since, first this is the most commonly used and available compound, which has all the necessary elements for SiCN coating formation as well as other bonds, and second, it has been shown that HMDS is safer to handle and provides obtaining of TiSiCN coating with a much lower friction coefficient than using tetramethylsilane [23].

Before the experiment, the specimens 4 of austenitic stainless steel AISI304 were cleaned in an ultrasound bath in acetone for 20 min, then dried and placed on an insulated holder, followed by a vacuum chamber pumped out to the residual pressure of 2 × 10^−2^ mTorr. Ar was the plasma-forming gas, but due to the low concentration of nitrogen in the HMDS molecule (8.7%), N_2_ was added to the gas mixture during deposition to optimise the elemental composition of produced coatings. Only Ar was supplied at the initial stage, with an impulse voltage of 3 kV supplied between the ignition electrode 5 and the cathode to ignite an additional discharge, which ignited the discharge glow between the hollow cathode and anode.

The ion bombardment of the inner surface of the cathode at a discharge current of 1 A heated it up to a temperature above 200 °C sufficient for effective thermoelectric mission [21], and the discharge started to burn at low voltage. The power supply used made it possible to adjust the current of discharge *I_d_* between 5 and 50 A, the voltage of discharge burning being 60–100 V.

Before the deposition cycle, specimens were ion-cleaned for 10 min by Ar ions at 500 eV. After cleaning, the energy was reduced to 70–100 eV. A share of the discharge current *I_cr_* was brought to the crucible before supplying voltage to it. The crucible temperature was adjusted by varying *I_cr_* within 0–10 A. Crucible temperature measurement showed that Ti melting was achieved at *I_cr_* = 1 A (power~200 W). By changing the *I_cr_* current in the crucible circuit from 1 to 10 A (power ~600 W), the flow of titanium vapours varied within ~10^−5^–10^−3^ g/s cm^2^, and the Ti deposition rate on the substrate respectively varied from 0.7 to 6 µm/h. To improve coating adhesion to the substrate, a 200–300-nm-thick Ti sub-layer was applied followed by precursor and nitrogen supply to the chamber. HMDS was supplied through the evaporator located 18 cm from the specimens. The vapour flow was adjusted by a Mini Cori-Flow digital mass flow controller (Bronkhorst) within 0–10 g/h.

The plasma composition was analysed using an OceanOptics HR2000 spectrometer (Ocean Optics inc., Orlando, FL, USA) within the wavelength range of 200 to 1100 nm, with a resolution of 0.84 nm. The coating thickness was measured by the abrasion of the specimen surface with a steel (VK6) ball in a Calotest instrument (CSM Instruments, Peseux, Switzerland) with an accuracy of up to 0.1 µm. The hardness (HV_0,02_) and Young’s modulus of the produced films were measured by a micro-indentation in the SHIMADZU DUH-211S at 20 mN. Averaging was done for 15–20 measurements. Measurements of X-ray photoelectron spectra (XPS) of specimens were recorded using PHI 5000 VersaProbe Scanning ESCA Microprobe spectrometer (Physical Electronics, Chanhassen, MN, USA) with monochromated Al Kα radiation, an X-ray beam was 200 µm. The energy resolution was better than 0.5 eV. The pressure in analytical chamber do not exceed 10^−7^ Pa during measurements. Ar^+^ ions (2 kV, 15 mA) sputtering was applied for surface clearing. The phase composition was studied by X-ray diffraction in a D8 DISCOVER diffractometer (Bruker AXS, Billerica, MA, USA) in copper Kα1 radiation using a graphite monochromator with a secondary beam. Diffraction patterns were processed using the TOPAS-3 software. The topology of coating irregularities was studied using a Tescan VEGA II XMU scanning electron microscope at 10^−5^ Torr with an INCA ENERGY 450 X-ray energy-dispersive microanalysis system. Adhesion tests were carried out using a Nanotest 600 device (Micromaterials Ltd., Wrexham, UK), the applied load was 7 N, the indenter radius is 50 microns. Averaging was carried out in five experiments.

## 3. Results and Discussion

One of the major problems in ensuring the stability of the discharge system during the production of organosilicon precursor coatings is that, during their plasma-chemical decomposition in the discharge, coatings with high dielectric properties are formed (except for hollow cathode) and the surface of the anode needs to be cleaned to ensure a stable combustion of the discharge and to prevent deterioration of the current-voltage characteristics thereof. In the absence of ion etching of the anode, the discharge voltage in the active gas medium containing OSC vapours gradually increases during the coating application process, indicating the formation of a dielectric film anode on the surface, which, in the end, could result in the discharge being extinguished.

**Figure 2:**
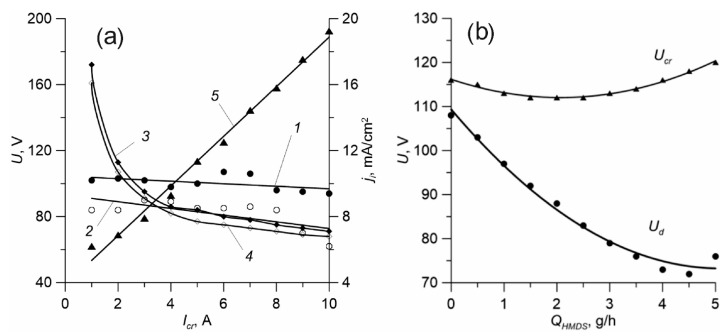
(**a**) Dependences of the main discharge voltage *U_d_* (1, 2), the voltage on the crucible *U_cr_* (3, 4) and the ion current density *j_i_* (5) on the current in the crucible circuit *I_cr_* without the precursor supply (1, 3) and with a flow of HMDS 1 g/h (2, 4). *Q_Ar_* = 40 sccm, *I_d_* = 20 A. (**b**) Dependences of the main discharge voltage *U_d_* and the voltage on the crucible *U_cr_* on the precursor flow. *Q_Ar_* = 40 cm^3^/min, *I_d_* = 20 A, *I_cr_* = 2 A.

Preliminary experiments have shown that the addition of titanium vapours to a vapour-gas mixture containing OSC causes the resultant coatings to become conductive and, when deposited on elements of the gas-discharge system, no deterioration in their electrical performance occurs. Thus, titanium vapours are a kind of stabilizing additive that ensures stable combustion of the discharge in a gas medium containing organosilicon precursor vapours. A wide range of discharge pressures with an SHHC allows to vary widely both the pressure of plasma-forming gases and the vapour flows of metal and precursor. At the same time, the increase in vapour pressure of the metal and the precursor has no significant effect on the stability of the gas discharge system. Introducing a crucible and titanium vapour supply into the discharge gap insignificantly affects the primary discharge burning (Figure 2a).

The increase of the current *I_cr_* from 1 to 10 A reduces the combustion voltage *U_d_* by only ~10–15 V, when the crucible is turned off, the *U_d_* is higher by 3–5 V. However, the voltage on the double layer of the volumetric spatial charge near the crucible that determines the *U_cr_* voltage between the cathode and the crucible is more dependent on the current in the crucible circuit. The voltage *U_cr_* varies from ~160–170 V to ~70 V within the *I_cr_* current range of 1–10 A. Such a rapid reduction of *U_cr_* is caused by a rapid increase in the pressure of saturated titanium vapours near the crucible as the temperature rises, and also by the low ionisation potential of metal atoms compared to plasma-forming gases (φ_Ti_ = 6.8 eV, φ_Ar_ = 15.7 eV, φ_N2_ = 15.5 eV).

Introducing precursor vapours reduces the voltage on the crucible and in the primary discharge gap, which can be explained by the overall increase in vapour-gas pressure at the discharge interval and the increase in the frequency of ionization. The increased flow of HMDS vapours affects *U_d_* to a greater extent than *U_cr_* (Figure 2b). This may be caused by the fact that *U_cr_* magnitude is defined mainly by the voltage drop on the double layer near the evaporated surface of the crucible, which, in turn, depends on the metal vapour pressure in the local area near the crucible, so the pressure growth of the gas mixture in the discharge gap does not significantly affect *U_cr_*. The ion current density *j_i_* to sample, with the full current of discharge *I_d_* from 10 to 50 A, linearly increased from 4 to 10 mA/cm^2^ without the crucible, and from 6 to 12 mA/cm^2^ at *I_cr_* = 2 A. The increase of the current of the crucible *I_cr_* to 10 A with the current of discharge *I_d_* = 20 A results in an increase of *j_i_* to almost 20 mA/cm^2^ (Figure 2a).

**Figure 3:**
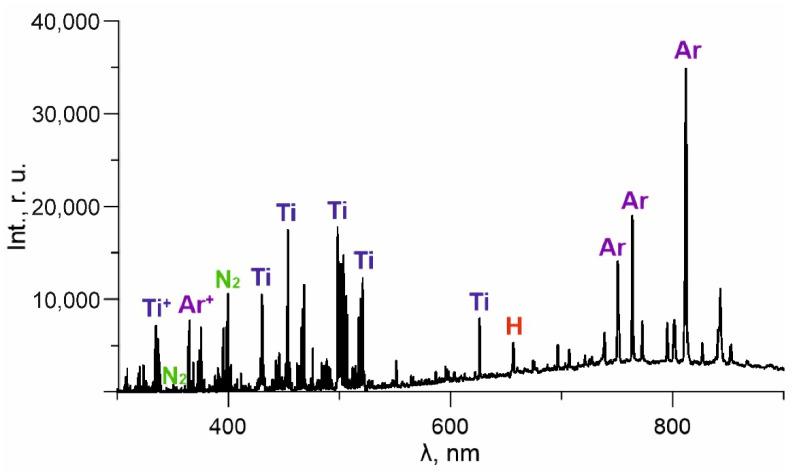
Optical spectra of discharge plasma.

The spectral analysis of plasma in the vapour and gas mixture containing vapours of titanium and HMDS showed that the medium had atoms and molecules of plasma-forming gases in excited and ionised states (Ar*, Ar^+^, N_2_*, N_2_^+^ 391.4 nm) and titanium atoms and ions (Ti*—399.9, 453.3, 498.2, 504 nm, Ti^+^—337.3, 364.3, 374.2 nm) (Figure 3).

**Figure 4:**
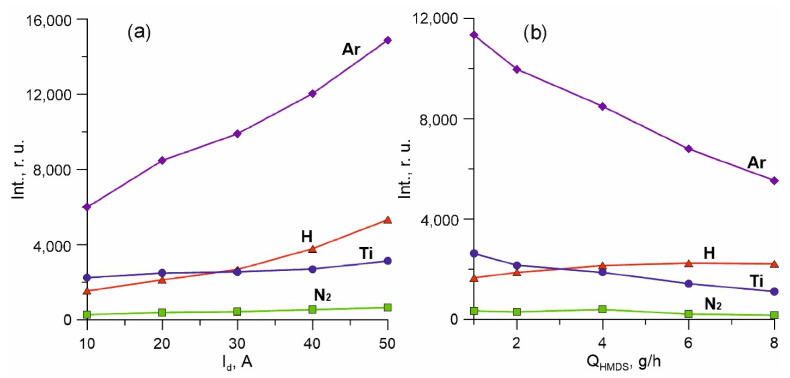
The dependences of the intensities of the spectral lines Ar (811.5 nm), Ti (453.3 nm), N_2_ (337.1 nm) and H (656.3 nm) on the discharge current *I_d_* (**a**) and on the *Q_HMDS_* flow (**b**). *I_cr_* = 2 A, *Q_Ar_* = 100 sccm, *Q_N2_* = 10 sccm. (**a**)—*Q_HMDS_* = 4 g/h, (**b**)—*I_d_* = 20 A.

Precursor decomposition products in the vapour gas mixture is evidenced by the presence of a strong line of the hydrogen atom (H* 656.3 nm) in the spectrum. However, no other HMDS element lines have been identified against the background of intense plasma-generating gas and titanium lines. An increase in current from 10 to 50 A results in a substantial increase in all gas lines of the spectrum, but the intensity of Ti lines is less dependent on *I_d_* (Figure 4). This may suggest that the metal vapour activation processes are predominantly in the crucible area and are much more dependent on *I_cr_* than *I_d_*. The intensity of line H* increases almost linearly (without saturation) with the current *I_d_*, which may indicate the presence of an excessive precursor and its incomplete decomposition within the specified range of currents.

At the same time, the increase in the vapour flow from 1 to 8 g/h at a direct current of discharge does not lead to an increase in the intensity of line H. This indicates a decrease in the average activation degree (i.e., the number of “activated” fragments in relation to the total number of initial molecules) and efficiency of using the precursor with the HMDS growing at a constant discharge current *I_d_*.

**Figure 5:**
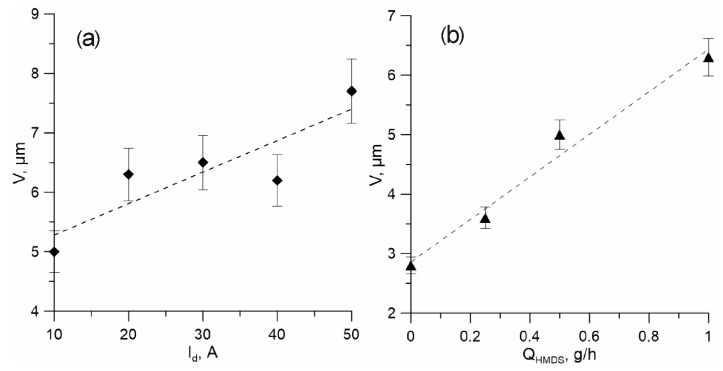
Dependences of the deposition rate of coatings on the discharge current *I_d_* (**a**) at *Q_HMDS_* = 1 g/h and on the flow Q_HMDS_ (**b**) at I_d_ = 20 A. I_cr_ = 2 A, P_tot_ = 0.45 mTorr.

Dependencies of coating growth rates on the discharge current and vapour flow are given in Figure 5. The deposition rate is increased monotonously from 5 to 7.5 µm/h as the discharge current grows within 10–50 A, and almost linearly depends on the HMDS flow when changed from 0 to 1 g/h.

Coatings for various discharge currents *I_d_* from 10 to 50 A were obtained, while other deposition conditions remained constant. The current in the crucible circuit was 2 A, the flow of HMDS vapours was 0.5 g/h, and the gas mixture pressure was 0.45 mTorr. Such deposition conditions were selected for test coatings based on preliminary experiments to ensure sufficiently high deposition rates of the coating (5–6 µm/h). The thickness of all produced coatings was 5–6 µm.

**Figure 6:**
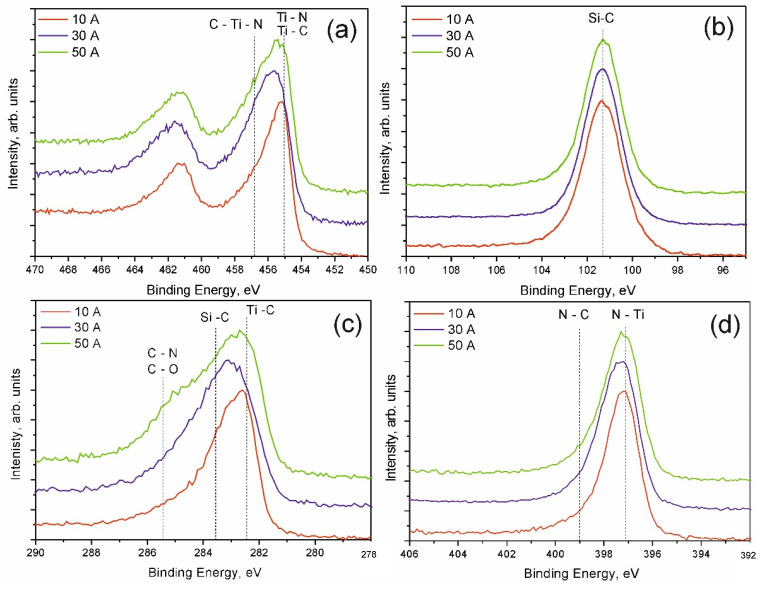
XPS spectra of TiSiCN coatings. Ti2p (**a**); Si2p (**b**); C1s (**c**); N1s (**d**).

The chemical composition and condition of bonds in the near-surface layer of specimens examined by XPS are given in Table 1 and Figure 6. The specimen produced at 10 A contains the highest amount of titanium (23.6 at.%), but also the least amount of nitrogen (19.3 at.%). As the discharge current grows from 10 to 50 A, the titanium concentration significantly falls from 23.6 to 16.9 at.%, while the nitrogen concentration rapidly grows from 19.3 to 27.9 at.%.

This may be explained by the fact that as the discharge current grows, the processes in plasma are intensified and the concentration of gas ions rises, in particular, molecular ions N_2_^+^, and, therefore, the concentration of atomic nitrogen [24] playing an important role in the formation of nitride coatings. The silicon concentrations in the obtained coatings (about 22 at.%) greatly exceeds optimal values of at.%, which is explained by the excessive and suboptimal flux of precursor vapours. Spectra of all Ti2p (a) specimens show areas corresponding to the formation of titanium chemical bonds with nitrogen (TiN), carbon (TiC) [25], and mixed bond C–Ti–N [26]. Spectra C1s (c) show stripes caused by the formation of Ti–C and Si–C, C–N, and C–O bonds [25]. Experimental data prove that carbon mainly forms bonds with titanium and silicone. An shoulder related to C–O bonds is found only in the spectrum of the specimen produced at 50 A. The composition of this specimen contains the highest amount of oxygen 5.1 at.%. The maximum position (101.3 eV) in Si2p (b) spectra shows that almost all silicon atoms chemically react with carbon forming Si-C bonds [25]. N1s (d) spectra show a strip with the maximum in the area of 397 eV ascribed to the Ti-N bond. The 399 eV area also shows a low-intensity component indicating nitrogen bonds with carbon.

**Figure 7:**
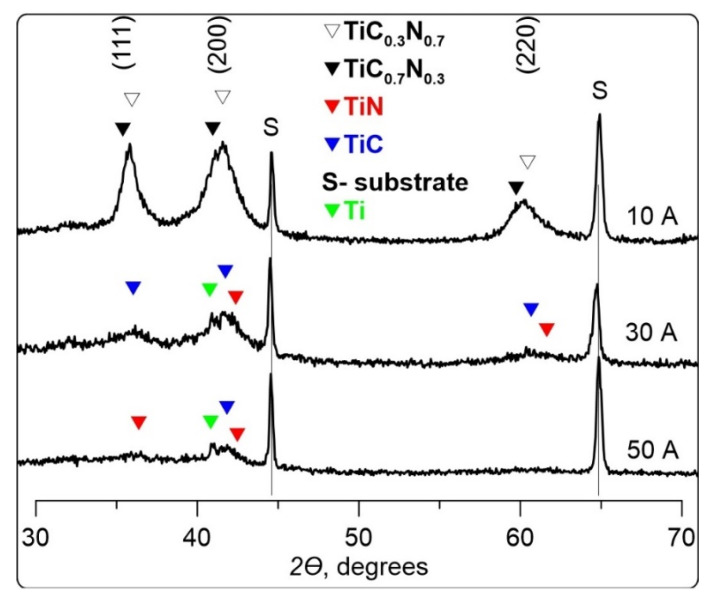
XRD patterns of TiSiCN coatings.

The results of the X-ray phase analysis are given in Figure 7. In the case of low discharge currents of 10–20 A, a solid solution based on TiCN is formed (phases TiC_0,3_N_0,7_ (PDF No. 42-1488, cubic, period a = 4.2644 Å) and TiC_0,7_N_0,3_ (PDF No. 42-1489, cubic, period a = 4.2971 Å) [14]). However, the observed phases of TiC_0,3_N_0,7_ (concentration 70%, coherent scattering region CSR ≈ 4.5 nm, period a = 4.330 Å) and TiC_0,7_N_0,3_ (concentration 22%, CSR ≈ 7 nm, period a = 4.388 Å) have significantly expanded lattice spacing as compared to literature data.

Possibly, this proves the presence of a solid solution based on these phases, and the lattice expansion might be caused by silicone atoms embedded into it. At elevated current (30–50 A) crystallite micro-structure consist of TiC (PDF No. 32-1383, cubic, period a = 4.327 Å) and TiN (PDF No. 38-1420, cubic, period a = 4.235 Å) [10]. The relative concentration of TiC/TiN falls from 12 to 0.25, the size of TiN crystallites decreases from 10 to 4 nm, and TiC crystallites are increased from 3 to 10 nm. Moreover, X-ray patterns show that as the current grows, the coatings become more X-ray amorphous, which may be explained by a reduced share of crystallite phases in the amorphous matrix. Crystallite phases TiSi, TiSiC, TiSi_2_, Si_3_N_4_, SIC are not found in any coating because Si might be present in the considered coatings only in amorphous phases of silicon carbide (a-Si_3_C_4_), silicon nitride (a-Si_3_N_4_), [27,28,29] and/or silicon carbonitride (a-SiC_x_N_y_) [7,30]. Overall diffraction patterns of TiSiCN coatings show the highest intensity in the diffraction plane (200).

**Figure 8:**
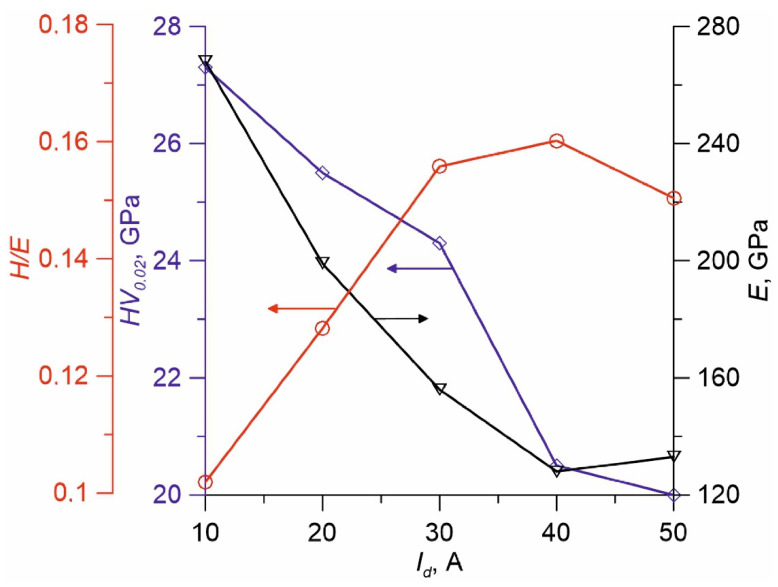
HV (blue), Young’s modulus (black) and H/E (red) of TiSiCN coatings on discharge current *I_d_*.

Figure 8 represents dependencies of coating hardness (HV), Young’s modulus E, and their relation H/E on the discharge current used to produce them. An increased discharge current from 10 to 50 A leads to a monotonous reduction of HV_0,02_ of coatings from 27.5 to 20 GPa, Young’s modulus falls from 260 to 130 GPa. The H/E ratio increases by more than 1.5 times from 0.1 to 0.16. Researchers [31,32] found that increased HV and impact strength of TiSiCN coatings resulted from the structural transformation into the nanocrystalline structure.

One of the primary advantages of using organosilicon precursors for the synthesis of nanocomposite coatings over other methods is the presence of some bonds in the precursor (Si–N, Si–C), so it is important to control the decomposition degree of some molecules during deposition, because an excessive power in the discharge may result in the breakdown of useful fragments. As the discharge current grows in the present experiments, the decomposition degree of the precursors rises in the gas phase when the plasma concentration increases and plasma-chemical processes are intensified in the volume. On the other hand, the density of ionic current rises along with the extent of the ion effect on the surface of the growing coating. Due to the excessive precursor in the plasma as the current increases, the OSC polymerisation process dominates on the surface, while crystalline formation slows down because of too low ratio of ions and neutrals *j_i_*/*j*_0_, even at high discharge currents. Possibly, HV reduction as current *I_d_* falls is related to a deeper decomposition of initial molecules of the precursor and decreased share of nc-phase in the coating, which is shown by a relative reduction intensity of lines on X-ray diffraction patterns (Figure 7).

Usually, H/E values for TiSiCN-coatings are 0.06–0.12 [33]. An increase in H/E values in the produced coatings as the discharge current grows can be possibly related to the increased concentration of the amorphous component of coatings as the precursor decomposition extent increases. The relatively low HV of produced coatings is probably related to the low *j_i_*/*j*_0_ on the substrate, since harder and denser coatings are formed at a higher level of ionic effect [31].

**Figure 9:**
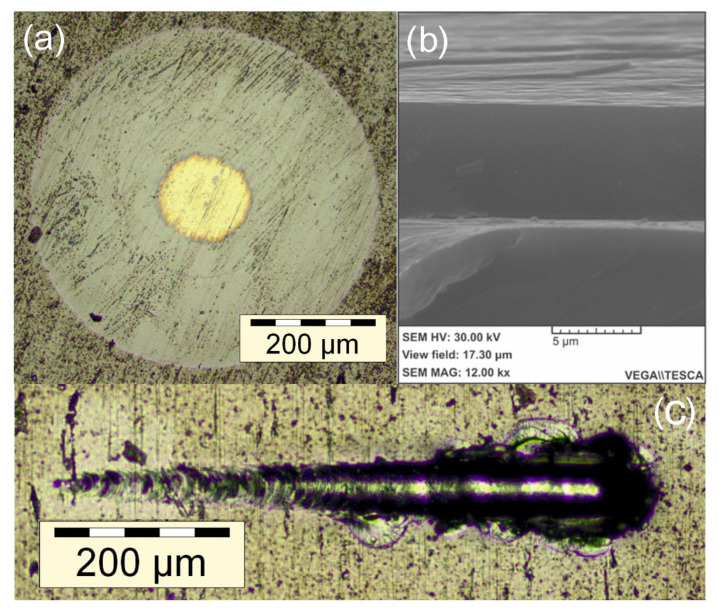
Indentation micrograph (**a**), cross section (**b**) and scratch test optical image (**c**) of TiSiCN film.

Images of the cross section (b) and the crater after ball abrasion (a) of the coating obtained at *I_d_* = 20 A, *I_cr_* = 2 A and *Q_HMDS_* = 1 g/h are shown in Figure 9. It shows that the coating structure is uniform and continuous (b), with no micro-structure found, which is aligned with data concerning the formation of the nanocomposite structure. No micro-drops on the coating surface are also found. Cracking of the coating along the edge of the indent is not observed (a). Good adhesion is consistent with high H/E values, which is an indicator of the resistance to cracking of the coatings. Result of scratch test of this sample is shown on Figure 9c. The point of destruction of the surface was determined visually and by the response from the acoustic emission sensor. The critical load *L_cr_*, the depth of penetration *h* of the indenter and the distance from the beginning of the scratch *d*, at which a stable destruction of the coating appears, were 3.9 N, 16.8 µm and 400 µm, respectively. With an increase in the *I_d_* current, the adhesion deteriorates significantly (*L_cr_* = 1.1 N, *h* = 3.7 µm, *d* = 110 µm), which may possibly be due to an increase in the proportion of the amorphous phase and, in particular, amorphous carbon, since amorphous carbon is usually brittle.

It should be noted that the obtained characteristics of coatings, in particular, elemental composition, HV, and H/E, are not optimal, therefore the optimisation of deposition and elemental composition to obtain the best performance coatings will be the subject of future research.

## 4. Conclusions

A new approach was developed to produce four-component nanocomposite coatings based on anodic evaporation of titanium and plasma-chemical decomposition of organosilicon precursor in the arc discharge with SHHC.

It has been demonstrated that it is possible to change both the composition of the vapour-gas mixture and, in particular, the ratio of vapour flows of the metal and the organosilicon precursor without affecting the operational stability of the discharge system and the density of the ion current, which allows a wide range of variations in the deposition rate of coatings, the degree of ion exposure and the active components of the vapour-gas medium.

It has been shown that this approach ensures a high evaporation rate of titanium (up to ~10^−3^ g/s cm^2^), activation of organosilicon precursor vapours and intensive ion support (up to *j_i_* ~20 mA/cm^2^) necessary to form hard TiSiCN-coatings at a high rate. The research team obtained nanocomposite TiSiCN-coatings with a dense homogeneous structure based on an amorphous SiCN matrix with embedded TiC, TiN, or TiCN crystals 3–10 nm in size, 20–27.5 GPa in density, H/E ratio up to 0.16 at the deposition rate up to 7–8 µm/h.

The proposed approach has a number of advantages; in particular, simple implementation, no additional devices both for creating the vapour flow of the metal and for filtering it, availability of accessible and affordable components for an active vapour-gas medium, and effective combinations of relatively high deposition rates and low energy consumption. All these advantages make the presented method attractive in terms of scaling and application within the industry.

## Figures and Tables

**Table 1:** The chemical composition of the surface of the samples (at.%) and the ratio of elements in the coatings.

*I_d_*, A	C	O	N	Si	Ti	C/Si	C/Ti	C/N	Si/N	Si/T
10	27.5	4.6	19.3	22.2	23.6	1.24	1.17	1.43	1.15	0.94
50	24.7	5.1	27.9	22.7	16.9	1.09	1.46	0.89	0.81	1.34

## Data Availability

Data is contained within the article.
